# Retraction: Hsia et al. Post-Intake of *S*-Ethyl Cysteine and *S*-Methyl Cysteine Improved LPS-Induced Acute Lung Injury in Mice. *Nutrients* 2016, *8*, 507

**DOI:** 10.3390/nu9060528

**Published:** 2017-05-23

**Authors:** Te-chun Hsia, Mei-chin Yin, Nutrients Editorial Office

**Affiliations:** 1 Department of Respiratory Therapy, China Medical University, Taichung City 40402, Taiwan; derrick.hsia@msa.hinet.net; 2 Department of Internal Medicine, China Medical University Hospital, Taichung City 40402, Taiwan; 3 Department of Nutrition, China Medical University, 91, Hsueh-shih Rd., Taichung City 40402, Taiwan; 4 Department of Health and Nutrition Biotechnology, Asia University, Taichung City 41354, Taiwan; 5 MDPI AG, St. Alban-Anlage 66, 4052 Basel, Switzerland; nutrients@mdpi.com

The integrity of several Western blot bands in Figures 3 and 4 [1] has been called into question. As a result, the authors of this article have decided to retract it and will repeat the entire analysis, to be submitted as a new article. We apologize to readers of *Nutrients* for any inconvenience caused.

*Nutrients* is a member of the committee on publication ethics (COPE) and takes the integrity of publications very seriously. In the interests of correcting the research literature, [1] will be marked as retracted.

## References

[1] Hsia T.-C., Yin M.-C. (2016). Post-intake of *S*-ethyl cysteine and *S*-methyl cysteine improved LPS-induced acute lung injury in mice. Nutrients.

